# Molecular Cloning and Functional Characterization of *TaWRKY65-3B*: Constitutive Overexpression in Rice Reduces Grain Yield and Reprograms Simulated-Drought-Responsive Metabolism via Diterpenoid Biosynthesis

**DOI:** 10.3390/antiox15101262

**Published:** 2026-10-01

**Authors:** Yanfei Zhang, Mengyuan Li, Gezi Li, Geng Ma, Haiyan Zhang, Lifang Wang, Wei Feng, Yingxin Xie, Wanzhang Wang, Dongyun Ma, Chenyang Wang

**Affiliations:** 1 College of Agronomy, Henan Agricultural University, Zhengzhou 450046, China15083380077@163.com (M.L.); ligezi@henau.edu.cn (G.L.); mageng@henau.edu.cn (G.M.); haiyanzhang@henau.edu.cn (H.Z.); lfwang8866@henau.edu.cn (L.W.); fengwei78@126.com (W.F.); xieyingxin@henau.edu.cn (Y.X.); 2 College of Mechanical & Electrical Engineering, Henan Agricultural University, Zhengzhou 450046, China; wangz@henau.edu.cn; 3 State Key Laboratory of High-Efficiency Production of Wheat-Maize Double Cropping, Henan Agricultural University, Zhengzhou 450046, China

**Keywords:** TaWRKY65-3B, transcriptome, metabolome, integrated analyses, simulated-drought stress

## Abstract

WRKY (WRKY domain-containing) transcription factors represent one of the largest families of plant-specific transcription regulators and play pivotal roles in plant growth, development, nutrient acquisition, and responses to biotic and abiotic stresses. In this study, the coding sequence of *TaWRKY65-3B* was cloned from the hexaploid wheat cultivar Hanxuan 10. Domain analysis revealed that TaWRKY65-3B contains a conserved WRKY domain and a typical C_2_H_2_-type zinc finger motif. The gene spans 1478 bp and comprises two exons and one intron. Promoter analysis identified multiple *cis*-acting regulatory elements, including those responsive to abscisic acid, methyl jasmonate, drought, and low temperature. Quantitative real-time PCR (qRT-PCR) demonstrated that *TaWRKY65-3B* was significantly upregulated under polyethylene glycol (PEG)-simulated-drought stress in wheat. Overexpression of *TaWRKY65-3B* in rice resulted in marked growth inhibition, characterized by reduced plant height, shortened grains, and decreased grain width, perimeter, and surface area. These morphological changes led to a significant reduction in thousand-grain weight and overall grain yield. Mechanistically, *TaWRKY65-3B* activated the expression of *OsCPS2*, *OsCPS4*, and *OsKS4*, which encode key enzymes in the diterpenoid phytoalexin biosynthetic pathway. Subsequent oxidative modifications catalyzed by the cytochrome P450 monooxygenase *OsCYP76M7* facilitated the accumulation of the defensive diterpenoid neoabietic acid. Integrated transcriptomic and metabolomic analyses revealed that constitutive *TaWRKY65-3B* overexpression reprograms simulated-drought-responsive metabolism in rice, with a diterpenoid metabolite annotated as neoabietic acid as a key correlative feature—though definitive structural confirmation requires authentic standards. These findings identify a WRKY-mediated regulatory module connecting diterpenoid phytoalexin metabolism to simulated-drought stress responses, although the precise molecular cascades governing the growth-defence trade-off between stress adaptation and yield warrant further investigation.

## 1. Introduction

Wheat is one of the most widely cultivated key food crops worldwide [1]. Wheat is primarily grown in arid and semi-arid regions, where unfavorable environmental factors such as drought, high temperatures, and low temperatures pose significant threats to sustainable production [2]. Against the backdrop of global warming and increasing frequency of extreme weather events, the adverse impacts of these conditions on wheat production are expected to intensify [3,4]. Therefore, given the current production environment and technological advancements, there is an urgent need to explore more excellent gene resources associated with high and stable yields.

WRKY (WRKY domain-containing) transcription factors primarily regulate the expression of downstream target genes by specifically binding to the *cis*-acting element W-box in the promoters [5]. They play roles in multiple growth and development processes [6,7,8] as well as in responses to various biotic [9,10,11,12] and abiotic stresses [13,14,15]. *AtWRKY14* acts as a suppressor of thermomorphogenesis in *Arabidopsis* [16]. In *Arabidopsis*, *AtWRKY42* regulates phosphorus homeostasis by modulating the expression of *AtPHO1* and *AtPHT1;1* to adapt to changes in phosphorus availability [17]. Similarly, *PdeWRKY6* and *PdeWRKY65* regulate phosphorus homeostasis by controlling the expression of *PdePHT1;9* in poplar [17,18]. ZmWRKY83 showed a continuous upregulation at both transcriptional and protein levels within 2 days after pathogen infection in maize, and overexpression of *ZmWRKY83* was found to enhance resistance to *Botrytis cinereain* in *Arabidopsis* [19]. *ZmWRKY104* improved salt tolerance by regulating the expression of *ZmSOD4* and increased superoxide dismutase activity in maize, which alleviated salt-induced increases in superoxide anion, malondialdehyde, and electrolyte leakage [20]. Additionally, ZmWRKY104 can be phosphorylated by ZmMPK6 and plays a role in ABA-induced antioxidant defense and drought tolerance in maize [21]. *ZmWRKY79* positively regulates the expression of phytoalexin biosynthesis genes in maize, functioning through the modulation of ABA biosynthesis under drought stress, and also participates in other abiotic stress responses [22]. The maize *wrky86* mutant exhibits enhanced tolerance to salt stress in plants [23]. In wheat, functions of a few members have been characterized. For instance, *TaWRKY10* and *TaWRKY44* act as positive regulators in drought, salt or osmotic stress responses by directly or indirectly activating the cellular antioxidant system to efficiently scavenge reactive oxygen species or by inducing the expression of stress-related genes, and overexpression of *TaWRKY10* and *TaWRKY44* enhances stress tolerance in transgenic tobacco [24,25]. The promoter activity of *TaWRKY2* can be induced by drought, salt, and ABA, and overexpression of *TaWRKY2* and *TaWRKY28* improved drought resistance in transgenic plants [26,27]. Overexpression of *TaWRKY1*, *TaWRKY10*, *TaWRKY33*, *TaWRKY46*, and *TaWRKY93* increased the maximum root length and enhanced stress resistance [24,28,29,30]. *TaWRKY51* promoted lateral root formation and yield-related traits while negatively regulating ethylene biosynthesis [31,32]. Although studies have shown that the *WRKY* family is large and functionally diverse, the function of *TaWRKY65-3B* has not yet been reported.

Drought is one of the most prevalent abiotic stresses that severely constrains plant growth, crop productivity, and geographical distribution worldwide [33]. To survive water-deficit conditions, plants have evolved sophisticated adaptive strategies, including stomatal regulation, osmotic adjustment, and the accumulation of reactive oxygen species (ROS)-scavenging antioxidants. Among these, the reprogramming of secondary metabolic pathways-such as the phenylpropanoid-flavonoid, terpenoid, and alkaloid biosynthetic routes-is a hallmark of plant acclimation to drought, as these metabolites function as antioxidants, UV protectants, and defensive compounds [34,35]. WRKY TFs are typically activated by drought-induced hormonal signals, particularly abscisic acid (ABA), jasmonic acid (JA), and salicylic acid (SA), as well as by MAPK cascades and ROS-mediated post-translational modifications [35]. Once activated, WRKY proteins bind specifically to W-box elements in the promoters of downstream target genes, where they can function as transcriptional activators or repressors, either independently or in concert with MYB-bHLH-WD40 (MBW) complexes [36]. Accumulating evidence has demonstrated that numerous WRKY members directly regulate key enzymes in secondary metabolic pathways-for example, phenylalanine ammonia-lyase (PAL), chalcone synthase (CHS), dihydroflavonol 4-reductase (DFR), 1-deoxy-D-xylulose-5-phosphate synthase (DXS), and tryptophan decarboxylase (TDC)-thereby modulating the accumulation of flavonoids, lignin, terpenoids, and alkaloids under drought stress [34,37]. For instance, studies in *Lycoris radiata* and mulberry (*Morus alba*) have revealed that specific WRKYs, such as *MaWRKY48*, play pivotal roles in integrating drought signals with flavonoid/anthocyanin biosynthesis [37,38]. Despite these advances, the precise WRKY-centered regulatory networks that link drought perception to the transcriptional reprogramming of secondary metabolism remain incompletely understood, particularly in non-model and economically important crops [36]. Elucidating how WRKY TFs mediate drought-induced secondary metabolite biosynthesis will not only deepen our understanding of plant stress-adaptation mechanisms but also provide valuable genetic targets for improving crop resilience and bioactive compound yields through molecular breeding or metabolic engineering.

In this study, transgenic rice lines overexpressing *TaWRKY65-3B* were generated to characterize their growth and agronomic performance. Integrated transcriptomic and metabolomic analyses were then conducted to investigate the molecular mechanisms underlying the drought-responsive metabolic reprogramming under simulated drought stress. This research provides a foundation for further elucidating the function of *TaWRKY65-3B* and understanding WRKY-mediated regulatory modules in cereal stress responses.

## 2. Materials and Methods

### 2.1. Plant Materials

Transgenic rice lines were generated as previously described with minor modifications [7]. The open reading frame (ORF) of *TaWRKY65-3B* (*TraesCS3B02G491100*) was amplified from the cDNA of the wheat cultivar “Hanxuan 10” using Pfu DNA polymerase. The amplified ORF was then cloned into the pCAMBIA1390 vector using a seamless cloning method, with the primers used listed in Table 1. This construct was subsequently transformed into the *Agrobacterium tumefaciens* strain EHA105. The recombinant *Agrobacterium* was then used to introduce the *TaWRKY65-3B* gene into rice (*Oryza sativa ssp. Japonica*, Kitaake), where its expression was driven by the ZmUbi promoter. The expression levels of *TaWRKY65-3B* in T3 generation homozygous lines were quantified by qRT-PCR. Based on the qRT-PCR results, two transgenic lines (OE15 and OE17) exhibiting different transcriptional levels of *TaWRKY65-3B* were selected for subsequent phenotypic analysis. All the plant materials mentioned above were provided by the Institute of Crop Science, Chinese Academy of Agricultural Sciences.

Germinated rice seeds (including wild-type control and two *TaWRKY65-3B* overexpression lines) were sown evenly in hydroponic trays and cultivated for two weeks in a growth chamber (Bluepard, Shanghai, China) under 30 °C (16 h light)/25 °C (8 h dark). The nutrient solution was renewed every other day to maintain uniform growth. Subsequently, plants were treated with either deionized water (control) or 192 g Polyethylene glycol 6000 (PEG6000)/kg H_2_O for simulated drought stress. PEG6000 (CP grade) was purchased from Sinopharm Chemical Reagent Co., Ltd. (SCR, Shanghai, China). The PEG6000 concentrations were calculated according to the equation previously reported [39], Ψs = − (1.18 × 10^−2^) C − (1.18 × 10^−4^) C^2^ + (2.67 × 10^−4^) CT + (8.39 × 10^−7^) C^2^T, where Ψs is the osmotic potential of the solution (bar, 1 bar = 0.1 MPa), C is the concentration of the PEG6000 solution (g/kg H_2_O), and T is the temperature of the solution (°C). At 25 °C, 192 g PEG6000/kg H_2_O yields Ψs ≈ −4.56 bar (−0.456 MPa), and at 30 °C, Ψs ≈ −4.15 bar (−0.415 MPa). This PEG6000-based hydroponic system, which imposes osmotic stress to simulate drought in rice and has been widely validated [40,41,42], was used at 192 g/kg H_2_O, a concentration where oxygen supply is not severely limiting according to the previously reported [43]. All groups shared identical transfer and handling conditions. Seedlings were grown in hydroponic tanks without active aeration for 3 days. The solution was completely replaced with freshly prepared solution every 24 h throughout the 3-day treatment to minimize solution stagnation. Shoots were sampled three days after treatment, rapidly frozen in liquid nitrogen, and stored at −80 °C for further analysis.

### 2.2. Subcellular Localization Assays

The subcellular localization assays were performed as previously reported [44], and the primers used were listed in Table 1.

### 2.3. Quantitative Real-Time PCR Assays

Total RNA was extracted using the Plant Total RNA Extraction Kit (R1050; Beijing Tianmo Technology Development Co., Ltd., Beijing, China). First-strand cDNA was synthesized using the FastQuant RT Kit (KR116; TIANGEN, Beijing, China) with 2 µg of total RNA. Quantitative real-time PCR (qRT-PCR) was performed using the SYBR^®^ Premix ExTaq™ Kit (RR420A; TaKaRa, Kusatsu, Japan) on an ABI QuantStudio^®^ 7 Flex Real-Time PCR System (Thermo Fisher Scientific, Waltham, MA, USA). qRT-PCR primers were designed based on the *TaWRKY65-3B*-specific sequence (*TraesCS3B02G491100*) and are listed in Table 1. Relative expression was normalized to *OsActin* (*LOC_Os03g50890*, for transgenic rice) and *TaActin* (*TraesCS2D02G491100*, for wheat). All qPCR reactions were performed in three technical replicates per biological replicate. Relative expression levels were calculated using the 2^−ΔΔCT^ method [44]. Data are presented as mean ± SD from three independent biological replicates.

### 2.4. Phenotyping of Transgenic Rice

For phenotyping, rice plants were grown in the paddy field of the Institute of Crop Sciences’ Experimental Station in Beijing (39°48′ N, 116°28′ E) during the 2019 and 2020 growing seasons. Agronomic traits, including plant height, root number, grain number per panicle, thousand-grain weight, and grain yield per plant, were measured at maturity. Harvested grains were analyzed for grain length, width, circumference, and area using a Wanshen SC-A grain counting and analysis system (Hangzhou Wanshen Testing Technology Co., Ltd., Hangzhou, China). All measurements were performed on at least three independent plants per genotype per year.

### 2.5. RNA Sequencing and Transcriptome Analysis

The transcriptomic assays were conducted by Shanghai Bioprofile Biotechnology Co., Ltd. (http://www.bioprofile.cn) (accessed on 23 June 2025). The steps were as follows:

#### 2.5.1. RNA Isolation and Library Preparation

Total RNA was isolated using TRIzol Reagent (Thermo Fisher Scientific, Waltham, MA, USA). RNA concentration, quality, and integrity were assessed using a NanoDrop spectrophotometer (Thermo Fisher Scientific, Waltham, MA, USA). Three micrograms of total RNA per sample were used as input material for sequencing library preparation. Sequencing libraries were generated for three independent biological replicates per group using the following steps.

mRNA was purified from total RNA using poly-T oligo-attached magnetic beads. Fragmentation was performed using divalent cations at elevated temperature in an Illumina proprietary fragmentation buffer. First-strand cDNA was synthesized using random oligonucleotides and SuperScript II reverse transcriptase. Second-strand cDNA synthesis was subsequently performed using DNA Polymerase I and RNase H. Remaining overhangs were converted into blunt ends via exonuclease/polymerase activities, followed by enzyme removal. After adenylation of the 3′ ends of the DNA fragments, Illumina paired-end (PE) adapter oligonucleotides were ligated. To select cDNA fragments of the preferred 400–500 bp in length, the library fragments were purified using the AMPure XP system (Beckman Coulter, Beverly, CA, USA). Fragments with ligated adapter molecules on both ends were selectively enriched using the Illumina PCR Primer Cocktail in a 15-cycle PCR reaction. The PCR products were purified with the AMPure XP system and quantified using the Agilent High Sensitivity DNA assay on a Bioanalyzer 2100 system (Agilent Technologies, Inc., Santa Clara, CA, USA). The resulting libraries were sequenced on a NovaSeq X Plus platform (Illumina, Inc., San Diego, CA, USA). Sequencing was performed with paired-end 150 bp reads, generating approximately 6 Gb of clean data per library (Q30 > 96%).

#### 2.5.2. Transcriptome Data Analysis

Quality Control: Raw sequencing data in FASTQ format were generated from image files produced by the sequencing platform. Adapters and low-quality reads were filtered out using Cutadapt (v1.15) to obtain high-quality clean reads for downstream analysis.

Read Mapping: The reference genome and corresponding annotation files were downloaded from the official genome database. Filtered reads were mapped to the reference genome using HISAT2 (v2.0.5).

Differential Expression Analysis: Read counts for each gene were obtained using HTSeq (v0.9.1) as the raw expression values, and expression levels were normalized to FPKM. Differential expression analysis was performed using DESeq (v1.30.0) with the following thresholds: |log_2_FoldChange| > 1 and adjusted *p*-value < 0.05. Bidirectional hierarchical clustering of differentially expressed genes (DEGs) was conducted using the R package pheatmap (v1.0.8). Euclidean distance and the complete linkage method were used to generate the heatmap. DEGs were identified from pairwise comparisons between OE15 and WT under both normal and PEG-simulated drought stress conditions, with the primary focus on OE15 vs. WT under PEG-simulated drought stress.

GO and KEGG Enrichment Analysis: All genes were annotated against the Gene Ontology (GO) database. GO enrichment analysis of DEGs was performed using topGO, with significance determined by the hypergeometric distribution (*p*-value < 0.05). KEGG pathway enrichment analysis was carried out using ClusterProfiler (v3.4.4), with significantly enriched pathways defined as those with *p*-value < 0.05.

### 2.6. Metabolome Analysis

Metabolome analysis was performed by Shanghai Bioprofile Biotechnology Co., Ltd. (http://www.bioprofile.cn) (accessed on 23 June 2025). The detailed procedures are as follows:

Sample preparation: Freeze-dried plant tissues were thawed at 4 °C and homogenized with pre-cooled methanol-water (4:1, *v*/*v*) using steel beads in a tissue homogenizer (−10 °C, 5 min). The homogenate was kept at −20 °C for 1 h and then centrifuged at 2300× *g* at 4 °C for 20 min. The supernatant was collected for LC-MS/MS analysis. Three biological replicates per treatment were used for metabolome analysis.

LC-MS/MS conditions: The supernatant was held at 4 °C in the autosampler prior to injection. Chromatographic separation was carried out on a Shimadzu Nexera X2 UHPLC system (Shimadzu Corporation, Kyoto, Japan) equipped with an ACQUITY UPLC HSS T3 column (2.1 × 100 mm, 1.8 μm) (Waters Corporation, Milford, MA, USA). The injection volume was 16 μL, the column temperature was maintained at 40 °C, and the flow rate was 300 μL/min. Mobile phases consisted of (A) 0.1% formic acid in water and (B) 0.1% formic acid in acetonitrile. The gradient elution program was as follows: 0 to 1 min, 2% B; 1 to 5 min, 2% to 48% B; 5 to 7 min, 48% to 80% B; 7 to 11 min, 80% to 100% B; 11 to 13 min, 100% B; 13 to 13.01 min, 100% to 2% B; 13.01 to 15 min, 2% B.

Mass spectrometry: Metabolites were analyzed using an AB SCIEX TripleTOF 6600 mass spectrometer (SCIEX, Framingham, MA, USA) with electrospray ionization in both positive (ESI+) and negative (ESI−) modes under information-dependent acquisition (IDA). The full-scan mass range was set to m/z 60 to 1200 Da, and the MS/MS mass range was m/z 25 to 1200 Da. Accumulation times were 0.08 s for MS and 0.03 s for MS/MS. Fifteen precursor ions were fragmented per cycle with a collision energy of 30 V ± 15 V. Source temperature was 500 °C; gas settings were GS1 = 60, GS2 = 60, CUR = 35; ion spray voltages were 5500 V (positive) and −4500 V (negative).

Data processing: Raw data were processed using MSDIAL (v4.9, RIKEN, Yokohama, Japan) for peak alignment, retention time correction, and peak-area extraction. Features with > 50% missing values in any group were discarded. Peak areas were normalized to total intensity separately for positive- and negative-ion data, merged, and scaled by unit variance (UV) in Python (v3.13.1, Python Software Foundation, https://www.python.org/, accessed on 23 October 2025) for subsequent statistical analysis. Metabolite identification was performed by accurate-mass matching (<10 ppm) and MS^2^ fragment matching (<0.01 Da) against the HMDB, MassBank, GNPS, and the in-house BP-DB library.

### 2.7. Integrated Transcriptomic and Metabolomic Analyses

KEGG pathway enrichment was performed separately for DEGs and DAMs as described above. Shared KEGG pathways were defined as the intersection of enriched pathway sets from the transcriptomic and metabolomic analyses. To focus on biologically relevant pathways showing coordinated (but not necessarily dual-significant) omics responses, we further applied a relaxed co-enrichment criterion within the shared pathway set: a shared KEGG pathway was retained as a co-enriched pathway if the enrichment *p*-value in either the transcriptomic dataset or the metabolomic dataset was *p* < 0.05 (*p*_DEG-pathway_ < 0.05 or *p*_DAM-pathway_ < 0.05).

### 2.8. Phylogenetic Tree Construction

The amino acid sequence of TaWRKY65-3B (XP_044344816.1) was used as a query to search the *Triticum aestivum* (IWGSC) database in the Ensembl Plants (https://plants.ensembl.org, accessed on 23 March 2025). Amino acid sequences of other reported WRKYs were obtained from the NCBI Protein database. The accession numbers of all WRKY protein sequences used for phylogenetic analysis and protein structure are listed in Supplementary Table S1. The evolutionary history was inferred using the maximum-likelihood method in MEGA7 (https://www.megasoftware.net/, accessed on 13 January 2026) with 1000 bootstrap replicates.

### 2.9. Cis-Acting Regulatory Elements Analysis

The promoter sequence of *TaWRKY65-3B* (*TraesCS3B02G491100*, 3000 bp upstream of the ATG start codon) was extracted from the *Triticum aestivum* reference genome (IWGSC RefSeq v1.1). Putative *cis*-acting regulatory elements within this region were predicted using the PlantCARE database (http://bioinformatics.psb.ugent.be/webtools/plantcare/html/, accessed on 23 October 2023).

### 2.10. Data Analysis

ANOVA followed by Duncan’s multiple range test was used to compare means using SPSS v.16.0. Graphics were displayed using GraphPad Prism 9 and Origin 2021.

## 3. Results

### 3.1. Analysis of TaWRKY65-3B Gene Sequence and Protein Structure

**Figure 1:**
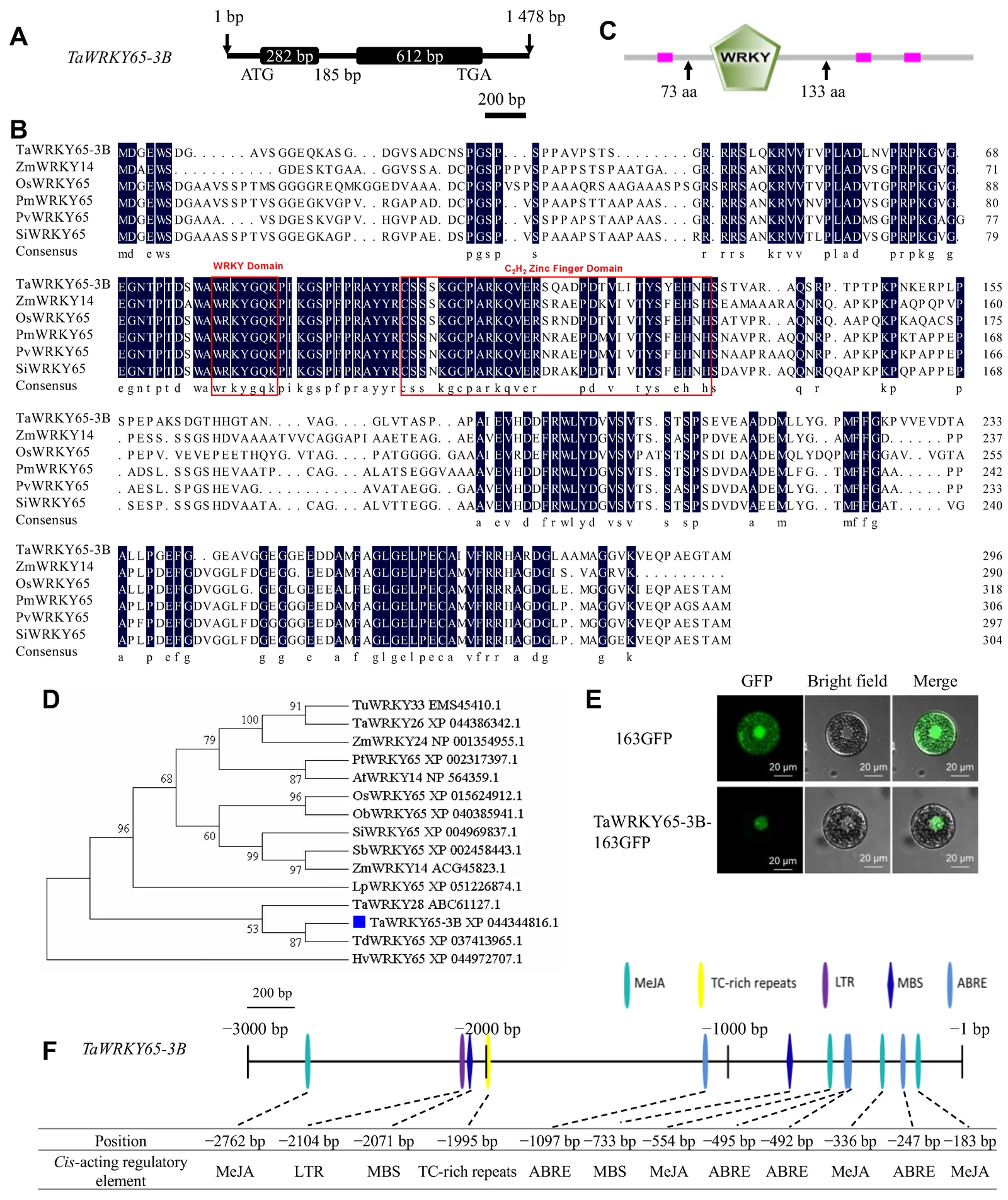
Analysis of the *TaWRKY65-3B* gene sequence and protein structure. (**A**) Diagram of the *TaWRKY65-3B* gene structure. Black rectangles indicate exons; (**B**) Alignment of TaWRKY65-3B proteins. The red rectangular box indicates the WRKY Domain and C_2_H_2_-finger domain. Accession numbers are provided in Supplementary Table S1; The black highlighted and letters below represent highly conserved sequences; (**C**) The prediction of TaWRKY65 secondary structure; (**D**) Phylogenetic tree of plant WRKYs. TaWRKY65-3B is labeled with a blue square. At, *Arabidopsis thaliana*; Hv, *Hordeum vulgare*; Lp, *Lolium perenne*; Ob, *Oryza brachyantha*; Os, *Oryza sativa*; Pt: *Populus trichocarpa*; Sb, *Sorghum bicolor*; Si, *Setaria italica*; Ta, *Triticum aestivum*; Td, *T. dicoccoides*; Tu, *T. urartu*; Zm, *Zea mays*; (**E**) Subcellular localization of TaWRKY65-3B in wheat protoplast cells; (**F**) Distributions of *cis*-acting regulatory elements in the 3000 bp promoter region (upstream of the ATG start codon) of *TaWRKY65-3B*, analyzed using the ‘Chinese Spring’ reference genome (IWGSC CS RefSeq v1.1, *GCA900519105.1*).

The *TaWRKY65-3B* (*TraesCS3B02G491100*) gene sequence was analysed using Ensembl Plants, which revealed that the genomic sequence was 1478 bp and comprised 2 exons and 1 intron. The coding region was 894 bp and encoded 297 amino acids (Figure 1A). The TaWRKY65-3B protein (XP_044344816.1) contains a conserved WRKY domain, which comprises a DNA-binding region and a C_2_H_2_-type zinc finger motif. Notably, TaWRKY65-3B possesses only one WRKY conserved domain (Figure 1B,C), and this domain is conserved across both monocot and dicot plants. Phylogenetic analysis showed that TaWRKY65-3B is highly conserved among cereal crops and is most closely related to TdWRKY65 and TaWRKY28 in wheat, which have been reported to be involved in drought stress responses, suggesting potential functional similarity (Figure 1D).

Subcellular localization demonstrated that TaWRKY65-3B exhibited nuclear localization in wheat protoplasts, a characteristic feature of transcription factors (Figure 1E). Analysis of the 3000 bp promoter sequence upstream of the ATG start codon identified multiple hormone-responsive and abiotic stress-responsive *cis*-acting elements, including the methyl jasmonate-responsive TGACG-motif and CGTCA-motif, the abscisic acid-responsive ABRE, the defense and stress-responsive TC-rich repeats, the low-temperature-responsive LTR, and the MYB-binding site (MBS) (Figure 1F). These results suggest that *TaWRKY65-3B* may be involved in the response to plant hormones such as methyl jasmonate and abscisic acid, as well as to osmotic and low-temperature stresses.

### 3.2. Overexpression of TaWRKY65-3B Represses Growth and Reduces Grain Yield in Transgenic Rice

**Figure 2:**
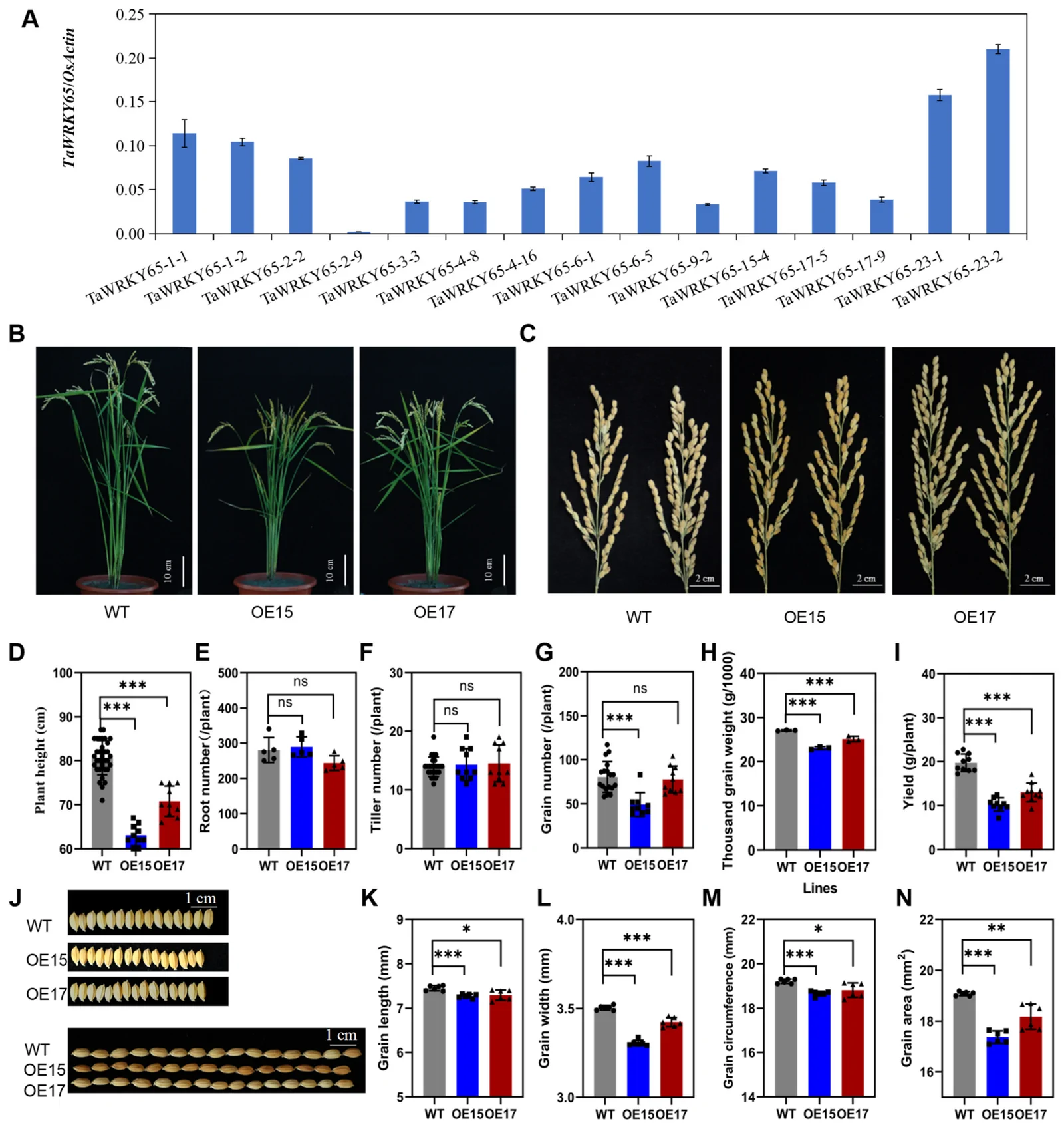
The effects of *TaWRKY65-3B* overexpression on the growth of rice. (**A**) The expression levels of *TaWRKY65-3B* in transgenic rice lines; Expression was normalized to *OsActin*; (**B**) Phenotypes of the rice plant at the mature stage; (**C**) Phenotypes of rice panicle at the mature stage; Comparisons of plant height (**D**), root number (**E**), tiller number (**F**), grain number (**G**), thousand grain weight (**H**) and grain yield per plant (**I**) between transgenics and the WT control; (**J**) Phenotypes of the grain; Comparisons of grain length (**K**), grain width (**L**), grain circumference (**M**), grain area (**N**); Data are given as means ± SD (from at least three independent samples) and were compared with WT by One-Way ANOVA in SPSS 16 (*, *p* < 0.05; **, *p* < 0.01; ***, *p* < 0.001; ns, no significant differences).

To investigate the impact of *TaWRKY65-3B* on growth and yield, we introduced the coding sequence (CDS) of *TaWRKY65-3B* into rice. Expression levels were quantified in T3 generation homozygous lines, and two homozygous overexpression lines, OE15 and OE17, were selected for further analysis (Figure 2A). It was shown that overexpression of *TaWRKY65-3B* significantly reduced plant height in rice at maturity (Figure 2B,D) but had no effect on root number (Figure 2E) or tiller number (Figure 2F). It was also shown that *TaWRKY65-3B* overexpression led to a decrease in the number of grains per panicle (Figure 2C). Notably, the grain number per panicle in OE15 was significantly lower than that in the wild type (WT) (Figure 2G). Overexpression of *TaWRKY65-3B* also led to a significant reduction in the thousand-grain weight (Figure 2H). Compared with WT (27.04 g), the thousand-grain weight of OE15 and OE17 was reduced by 4.01 g (14.8%, to 23.03 g) and 1.95 g (7.20%, to 25.09 g), respectively. As a result, grain yield per plant also decreased (Figure 2I), with the overexpression lines showing reductions of 9.45 g/plant in OE15 (47.9%, to 10.27 g/plant) and 6.71 g/plant in OE17 (34.0%, to 13.01 g/plant) compared with WT (19.72 g/plant). Further analysis of grain size revealed that *TaWRKY65-3B* overexpression significantly decreased the grain length (Figure 2J,K), grain width (Figure 2J,L), grain circumference (Figure 2M), and grain area (Figure 2N).

### 3.3. RNA-Seq Analysis of TaWRKY65-3B in Rice Under PEG-Simulated Drought Stress

#### 3.3.1. TaWRKY65-3B Mediates Phenotypic Responses and Transcriptomic Changes in Rice Under PEG-Simulated Drought Stress

**Figure 3:**
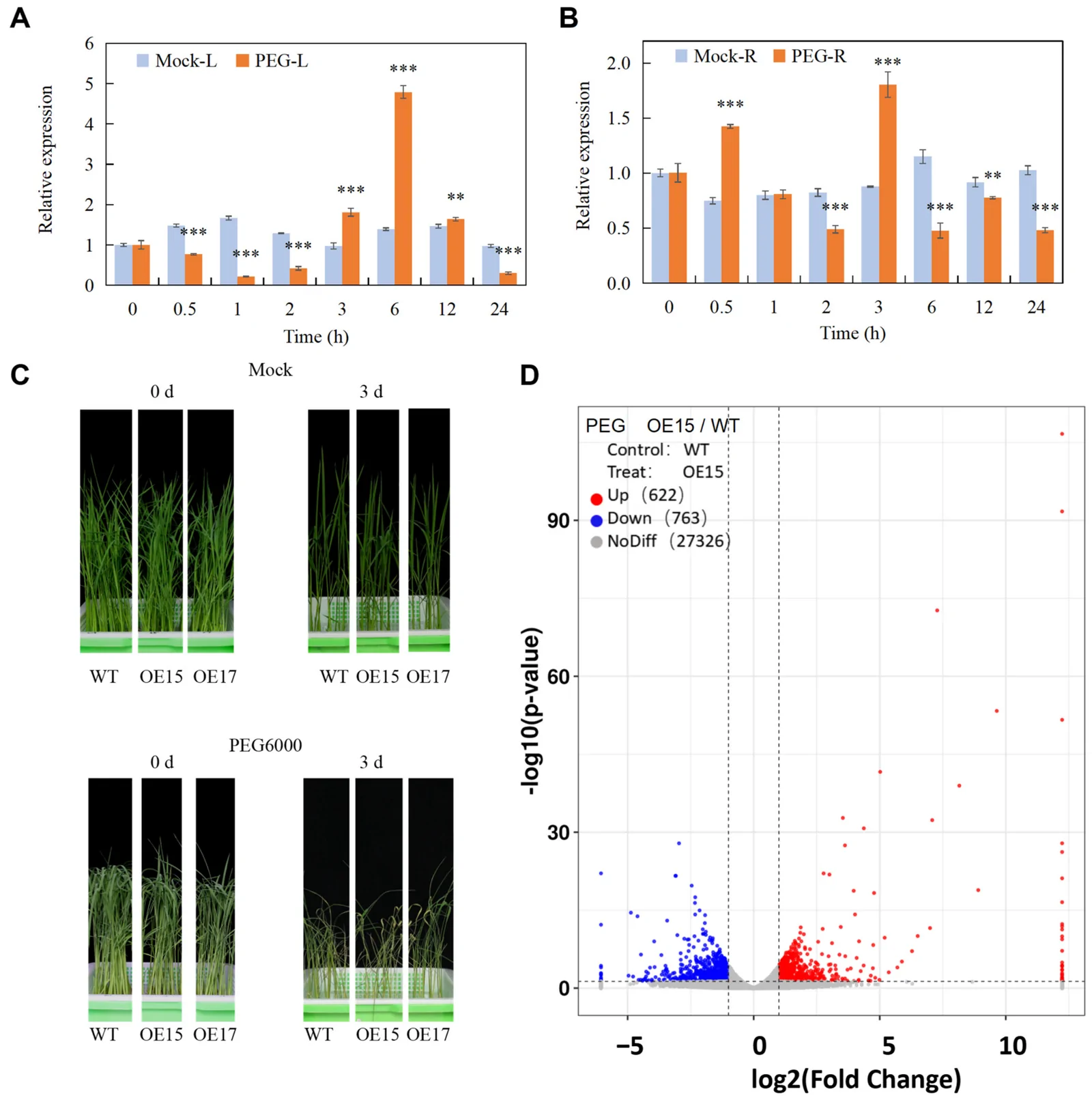
The phenotype and the number of differentially expressed genes under PEG-simulated drought stress. (**A**,**B**) Expression patterns of *TaWRKY65-3B* under PEG-simulated drought stress in leaves and roots of wheat; Expression was normalized to *TaActin*; Data are given as means ± SD (from at least three independent samples) and were compared with WT by One-Way ANOVA in SPSS 16 (**, *p* < 0.01; ***, *p* < 0.001). (**C**) Remaining seedlings after destructive sampling at each time point under mock and PEG-simulated drought stress; (**D**) The number of differentially expressed genes under PEG-simulated drought stress. Red indicates up-regulated expression, and blue indicates down-regulated expression in WT;. Two vertical dashed lines indicate the 2-fold expression difference threshold; the horizontal dashed line indicates the *P*-value = 0.05 threshold.

The promoter region of *TaWRKY65-3B* contains multiple hormone-responsive and abiotic stress-responsive *cis*-acting elements. To investigate the role of *TaWRKY65-3B* in abiotic stress responses, we first analyzed its expression pattern in wheat seedlings under PEG-simulated drought stress. The results showed that the expression levels of *TaWRKY65-3B* in leaves and roots peaked at 6 h and 3 h after treatment, respectively, reaching 4.79-fold and 1.80-fold that of the pre-treatment levels (Figure 3A,B). To further explore the mechanism by which *TaWRKY65-3B* participates in drought stress, transgenic rice overexpressing *TaWRKY65-3B* (OE15 and OE17) was subjected to PEG6000 treatment. After 3 days of PEG treatment, the overexpression lines and WT plants exhibited distinct morphological responses, including differential degrees of leaf curling and tip wilting (Figure 3C). The apparent wilting and slight leaf tip desiccation observed in the water controls were primarily procedural artifacts resulting from brief exposure to ambient air with different temperature and humidity during temporary removal from the controlled-environment growth chamber for photography.

Given the consistent phenotypic trends observed between the two independent lines and cost constraints, OE15 was selected as the representative line for subsequent transcriptomic and metabolomic profiling. Using the criteria of |log_2_FoldChange| > 1 and adjusted *p*-value < 0.05, RNA-seq analysis identified a total of 1385 DEGs under PEG-simulated drought stress. Among the 1385 DEGs identified in OE 15 vs. WT, 622 DEGs were more highly expressed in PEG-treated OE15 plants and 763 DEGs were more highly expressed in WT (|log_2_FC| ≥ 1, *p* < 0.05). (Figure 3D). The broader distribution of expression changes observed among the up-regulated genes suggests that *TaWRKY65-3B* may play a role in stress adaptation by modulating the expression of these up-regulated genes.

#### 3.3.2. GO Enrichment Analysis of the DEGs Under PEG-Simulated Drought Stress

**Figure 4:**
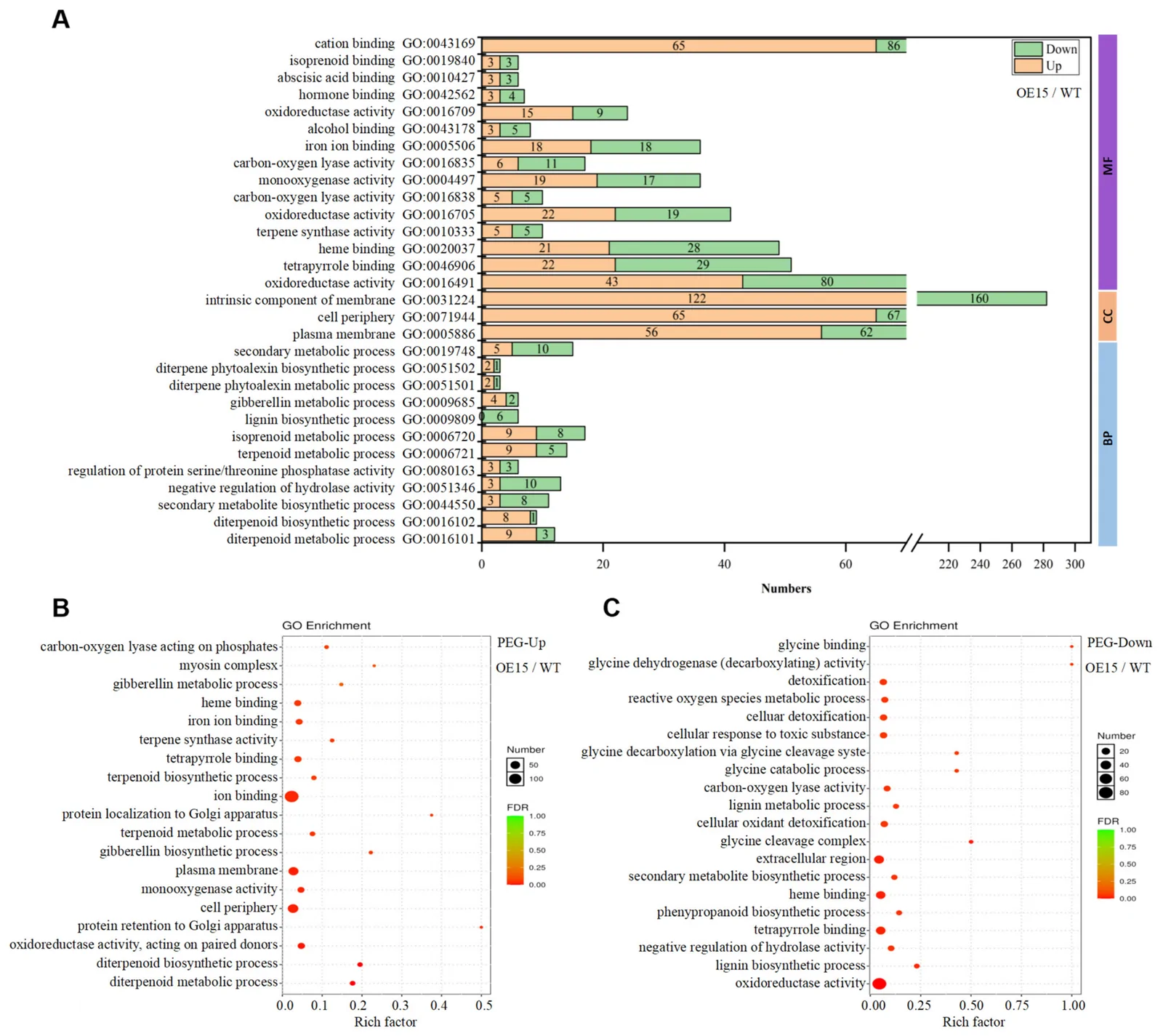
The GO enrichment analysis of differentially expressed genes under PEG-simulated drought stress. (**A**) The number of differentially expressed genes in the top 30 most significantly enriched GO terms; (**B**) Bubble plot of the top 20 most significantly enriched GO terms of the upregulated differentially expressed genes; (**C**) Bubble plot of the top 20 most significantly enriched GO terms of the downregulated differentially expressed genes. A larger Rich factor indicates a greater degree of enrichment. The FDR (False Discovery Rate) generally ranges from 0 to 1, and a value closer to zero indicates more significant enrichment.

All DEGs were functionally annotated using GO. A total of 1133, 1263, and 1860 DEGs were annotated to the categories of biological process (BP), cellular component (CC), and molecular function (MF) under PEG-simulated drought stress, respectively. The most significantly enriched BP terms included diterpenoid metabolism and biosynthesis, diterpenoid phytoalexin metabolism and biosynthesis, isoprenoid metabolism, and secondary metabolism process. The main enriched CC terms were plasma membrane, cell periphery, and intrinsic components of membrane. The predominant MF terms comprised oxidoreductase activity, terpene synthase activity, monooxygenase activity, carbon-oxygen lyase activity, and ion binding (Figure 4A). These results suggest that *TaWRKY65-3B* may be involved in regulating the synthesis and metabolism of diterpenoids, redox reactions, and ion binding under PEG-simulated drought stress.

GO enrichment analysis revealed that up-regulated genes were primarily associated with protein localization and retention in the Golgi apparatus, as well as gibberellin and diterpenoid biosynthesis and metabolism (Figure 4B). The Golgi apparatus functions in protein processing, sorting, and transport; the retention of proteins therein may facilitate targeted modifications under drought stress. For gibberellin-related pathways, dynamic regulation contributes to stress adaptation by balancing resistance and growth, potentially through mediating osmolyte synthesis, antioxidant enzyme activity, cell wall modification, and hormonal crosstalk with ABA, CTK, and IAA. Conversely, down-regulated genes were significantly enriched in glycine catabolism (Figure 4C). Glycine breakdown, driven by enzymes such as the glycine decarboxylase complex, releases energy to support ion transport, osmolyte synthesis, and antioxidant activation, thereby mitigating reduced photosynthetic efficiency. Intermediate metabolites (e.g., glyoxylate and serine) can enter the TCA cycle or gluconeogenesis, supplying precursors for carbohydrates and amino acids to sustain biosynthesis and prevent metabolic arrest under stress. These enrichment patterns provide insights into the potential mechanisms by which *TaWRKY65-3B* coordinates metabolic and hormonal responses under PEG-simulated drought stress.

#### 3.3.3. KEGG Pathway Enrichment of DEGs Under PEG-Simulated Drought Stress

**Figure 5:**
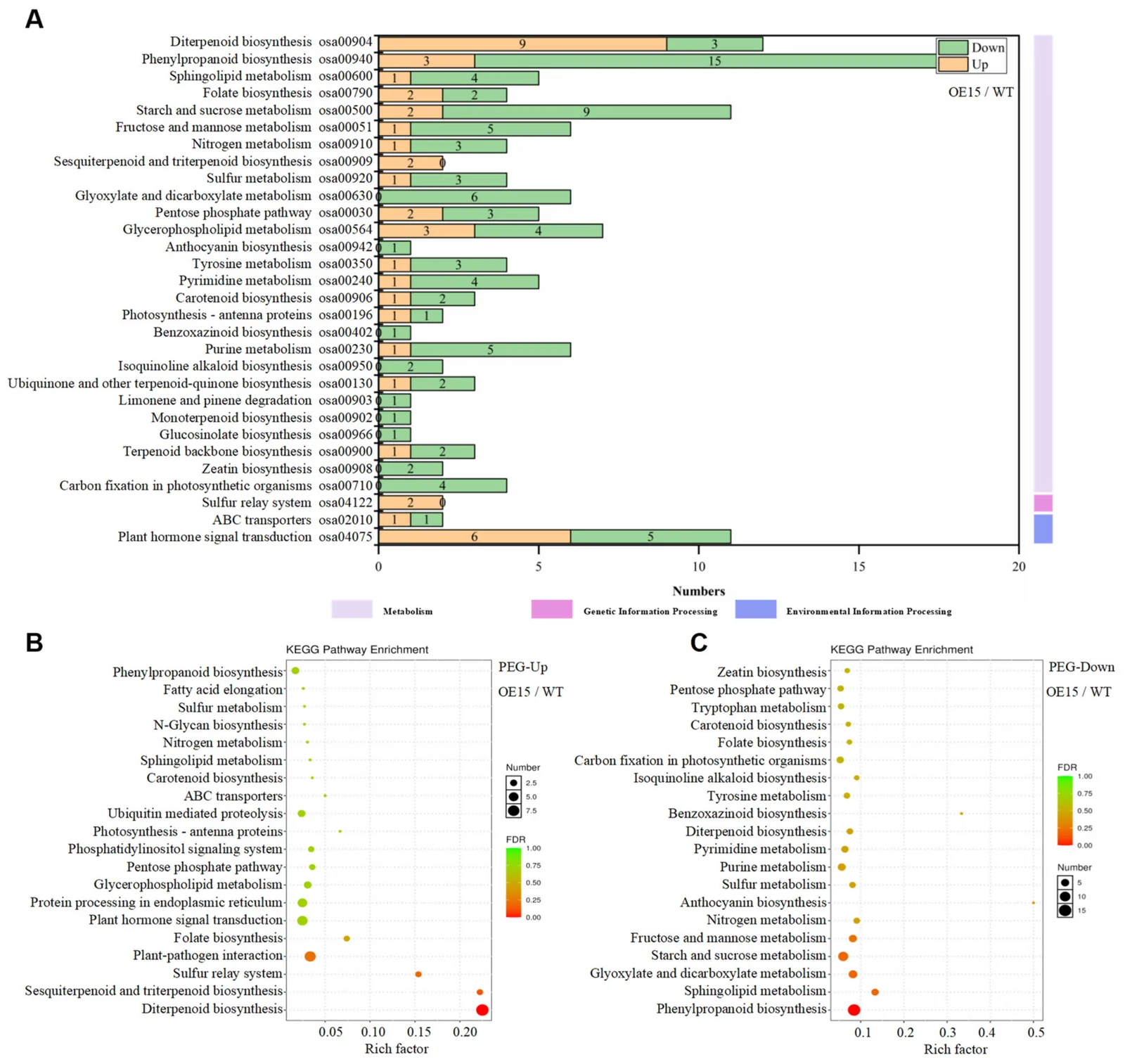
The KEGG enrichment analysis of differentially expressed genes under PEG stress treatment. (**A**) The number of differentially expressed genes in the top 30 most significantly enriched KEGG terms; (**B**) Bubble plot of the top 20 most significantly enriched pathways of the upregulated differentially expressed genes; (**C**) Bubble plot of the top 20 most significantly enriched pathways of the downregulated differentially expressed genes. A larger Rich factor indicates a greater degree of enrichment. The FDR (False Discovery Rate) generally ranges from 0 to 1, and a value closer to zero indicates more significant enrichment.

KEGG pathway enrichment analysis of the DEGs identified 89 significantly enriched pathways, which were classified into five major categories: cellular processes (4 DEGs), environmental information processing (21 DEGs), genetic information processing (25 DEGs), metabolism (204 DEGs), and organismal systems (9 DEGs) (Figure 5A). Among the top 30 most significantly enriched pathways, metabolism-related pathways—including terpenoid backbone biosynthesis, phenylpropanoid biosynthesis, and starch and sucrose metabolism—as well as environmental information processing-related pathways, such as ABC transporters and plant hormone signal transduction, were predominantly represented. Further analysis of regulation direction revealed that up-regulated DEGs were primarily enriched in the terpenoid backbone biosynthesis pathway (Figure 5B), whereas down-regulated DEGs were significantly enriched in the phenylpropanoid biosynthesis pathway (Figure 5C).

### 3.4. Metabolomic Analysis of TaWRKY65-3B Involvement in PEG-Simulated Drought Stress

**Figure 6:**
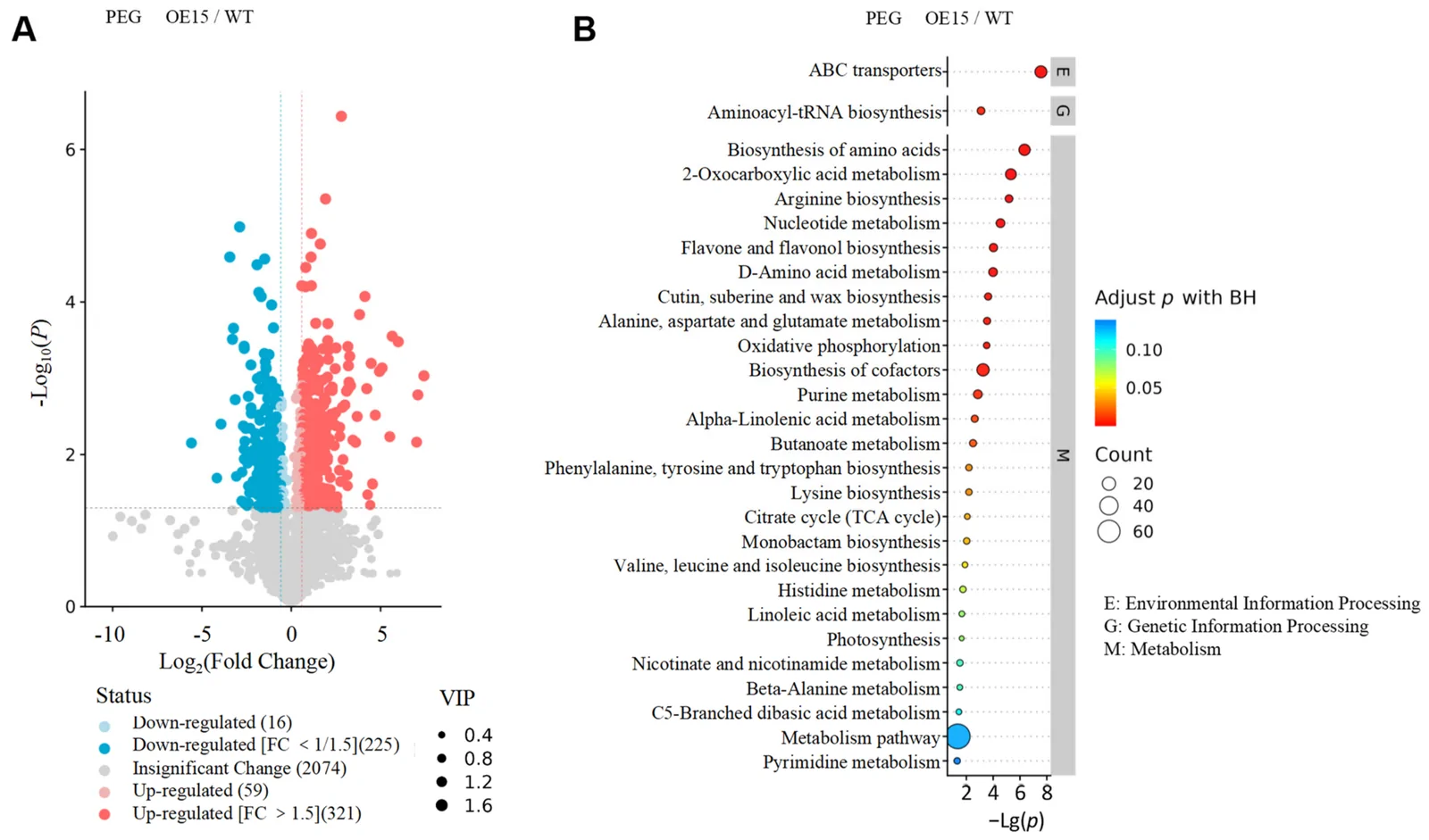
Volcano plot and KEGG pathway enrichment bubble plot of differential metabolites under PEG-simulated drought stress. (**A**) Volcano plot of differential metabolites in OE15 relative to WT under PEG-simulated drought stress. The *x*-axis represents log_2_(fold change) and the *y*-axis represents −log_10_(adjusted *p*-value) (Benjamini–Hochberg FDR); Vertical dashed lines indicate the fold-change thresholds (Log_2_(Fold Change) corresponding to FC = 1/1.5 and FC = 1.5). The horizontal dashed line indicates the significance threshold (*P*-value = 0.05; −log_10_(*P*) ≈ 1.3). Red points, up-regulated; blue points, down-regulated; gray points, insignificant change. (**B**) KEGG pathway enrichment bubble plot of differential metabolites. Bubble size indicates the number of enriched metabolites; bubble color indicates the enriched significance level (−log_10_(adjusted *p*-value)).

Plant metabolites play crucial roles in responses to both biotic and abiotic stresses, acting as osmoregulators, osmoprotectants, and signaling molecules. Compared with genes and proteins, metabolites are closer to the phenotypic level and thus more directly reflect the integrated outcomes of gene expression and regulatory networks. Metabolomic profiling provides a powerful approach for comprehensively uncovering physiological and biochemical changes in plants. To investigate metabolic alterations in rice overexpressing *TaWRKY65-3B*, UPLC-MS/MS was employed to compare the metabolomes of transgenic and WT plants under normal and PEG-simulated drought conditions. Under PEG-simulated drought stress, a total of 2695 annotated metabolites were identified (Figure 6A), among which 546 DMs were detected based on VIP ≥ 1, |FC| ≥ 1.5, and *p* < 0.05. Of these, 321 were up-regulated and 225 were down-regulated in OE15.

KEGG pathway enrichment analysis of DMs revealed significant enrichment in several key metabolic pathways, including ABC transporters, aminoacyl-tRNA biosynthesis, amino acid biosynthesis and metabolism, flavonoid and flavone biosynthesis, purine and pyrimidine metabolism, the tricarboxylic acid (TCA) cycle, oxidative phosphorylation, pectin/cutin/suberin wax biosynthesis, α-linolenic acid metabolism, and linoleic acid metabolism (Figure 6B). Under PEG-simulated drought stress, plants likely mobilize amino acid and nucleotide metabolism as well as energy metabolism to support cellular homeostasis and macromolecule synthesis. Concurrently, the flavonoid biosynthesis pathway is activated as a defensive response, while ABC transporters may facilitate the transport of energy substrates and stress-related compounds.

### 3.5. Integrated Transcriptomic and Metabolomic Analyses of TaWRKY65-3B Involvement in PEG-Simulated Drought Stress

**Figure 7:**
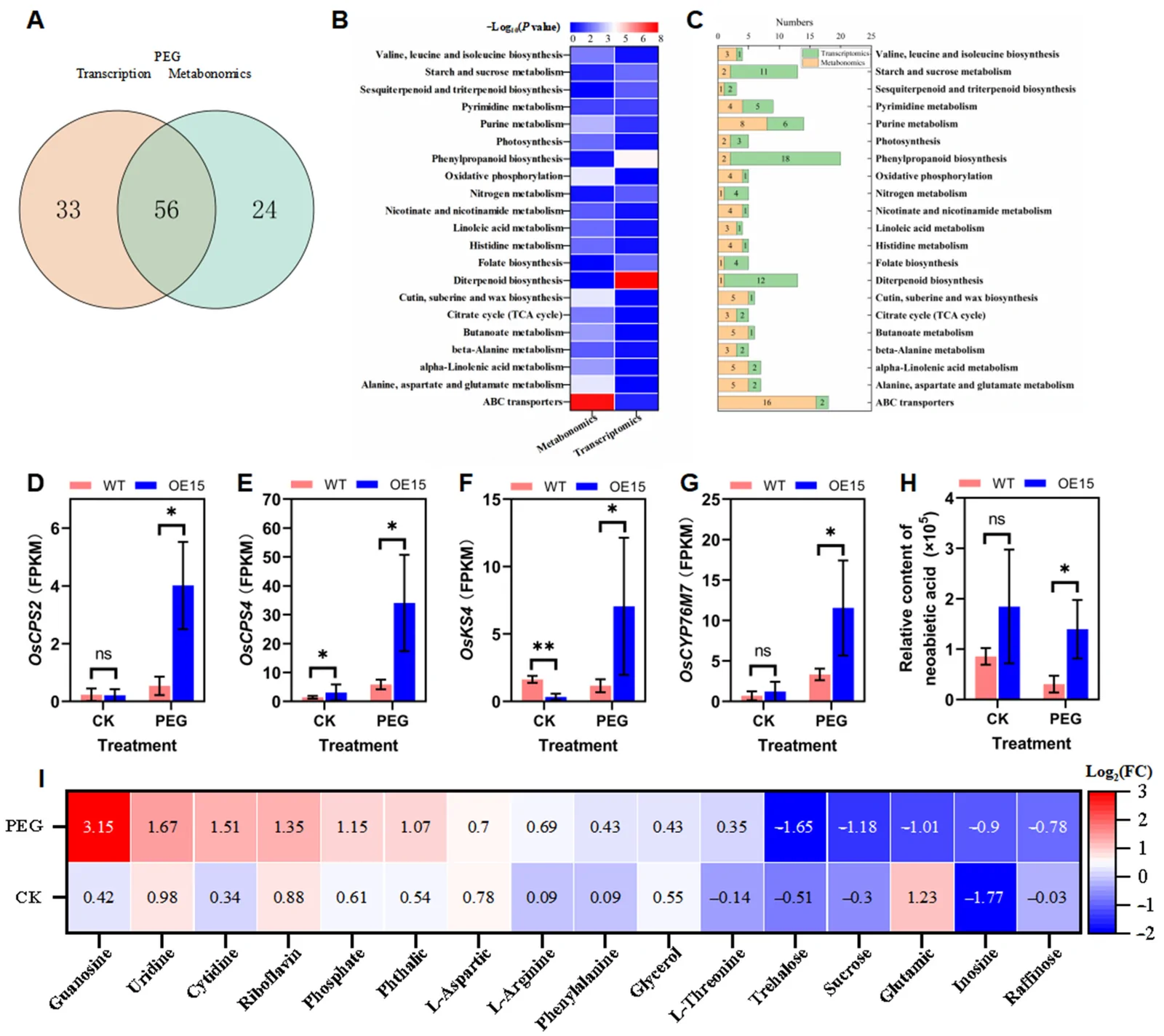
Combined transcriptomics and metabolomics analysis under PEG-simulated drought stress. (**A**) Number of shared KEGG pathways between transcriptome and metabolome under PEG-simulated drought stress; (**B**) Heatmap of significance for co-enriched KEGG pathways. Color corresponds to −log_10_(*p*-value), with redder color indicating higher significance; (**C**) Number of differential metabolites or genes annotated to the co-enriched KEGG pathways; (**D**–**G**) Transcriptional levels of up-regulated genes in the diterpene biosynthesis pathway; Data are given as means ± SD (from at least three independent samples) and were compared with WT by One-Way ANOVA in SPSS 16 (*, *p* < 0.05; **, *p* < 0.01; ns, no significant differences). (**H**) The relative content of neoabietic acid under CK and PEG-simulated drought stress; (**I**) Fold changes (log_2_ scale) of 16 differential metabolites in the ABC transporters pathway. Eleven up-regulated and five down-regulated metabolites in OE15 relative to WT under PEG treatment are indicated. Note: The metabolite name reflects database annotation, not standard-confirmed identification.

Integrated transcriptomic and metabolomic analyses refer to the combined investigation of gene expression and metabolite profiles to elucidate their relationships within biological systems. KEGG pathway-based joint analysis of the transcriptomic and metabolomic data revealed that, under PEG-simulated drought stress, 56 KEGG pathways were co-enriched across both datasets (Figure 7A), of which 21 were significantly enriched in both (Figure 7B). The most prominently enriched pathway in the transcriptome was diterpenoid biosynthesis, which comprised 12 related genes (Figure 7C). Among these, 9 were up-regulated—namely *Os04g0178400*, *Os06g0569500* (*OsKO4*), *Os06g0568600* (*OsKO5*), *Os02g0571100* (*OsCPS2*) (Figure 7D), *Os04g0178300* (*OsCPS4*) (Figure 7E), *Os04g0179700* (*OsKS4*) (Figure 7F), *Os02g0570400* (*OsKS7*), *Os02g0630300* (*OsGA2ox9*), and *Os02g0569900* (*OsCYP76M7*) (Figure 7G)—and 3 were down-regulated: *Os06g0599200*, *Os05g0514600* (*OsGA2OX4*), and *Os05g0560900* (*OsGA2ox8*). Correspondingly, the diterpenoid biosynthesis pathway was also enriched in the metabolome. Among the differential metabolites, a diterpenoid compound was annotated as neoabietic acid based on MS/MS spectral matching, without confirmation by an authentic standard (Figure 7C).

PEG-simulated drought stress reduced the relative content of this metabolite (normalized peak area) in WT plants. However, overexpression of *TaWRKY65-3B* partially rescued this reduction, leading to a significant increase in its levels in OE15 under PEG-simulated drought stress (FC = 2.195, *p* < 0.05 vs. WT; Figure 7H). These results suggest that, under PEG-simulated drought stress, the accumulation of this diterpenoid metabolite is associated with the up-regulation of key diterpenoid biosynthetic genes, including *OsCPS2*, *OsCPS4*, and *OsKS4*, which are involved in diterpene skeleton formation. The concomitant induction of *OsCYP76M7*, a cytochrome P450 monooxygenase implicated in rice phytocassane biosynthesis, further supports activation of the diterpenoid phytoalexin pathway. Given that these genes are well-characterized components of the phytocassane/momilactone biosynthetic network, the metabolite annotated as neoabietic acid may represent a phytocassane-type diterpenoid or a structurally related compound within this pathway. Further biochemical validation with authentic standards is required to confirm the exact structure of this metabolite and the direct catalytic roles of the associated enzymes.

The highest enrichment degree in the metabolome was observed for ABC transporters (Figure 7B), corresponding to 16 differential metabolites (Figure 7C). Among these, 11 were up-regulated, including guanosine (log_2_FC = 3.148), uridine (log_2_FC = 1.673), cytidine (log_2_FC = 1.508), riboflavin (log_2_FC = 1.352), phosphate (log_2_FC = 1.147), phthalic acid (log_2_FC = 1.071), L-aspartic acid (log_2_FC = 0.695), L-arginine (log_2_FC = 0.686), phenylalanine (log_2_FC = 0.429), 3-phosphoglycerate (log_2_FC = 0.428), and L-threonine (log_2_FC = 0.348); 5 were down-regulated, including trehalose (log_2_FC = −1.654), sucrose (log_2_FC = −1.181), glutamic acid (log_2_FC = −1.005), inosine (log_2_FC = −0.897), and raffinose (log_2_FC = −0.783) (Figure 7I). These results suggest that, under PEG-simulated drought stress, ABC transporters are involved in the transmembrane transport of these metabolites, contributing to osmotic adjustment and cellular homeostasis.

Based on the above analysis, PEG-simulated drought stress triggers a coordinated metabolic response in plants: the diterpenoid biosynthesis pathway is activated, with a metabolite annotated as neoabietic acid identified among the differential compounds; concurrently, the ABC transporter pathway is enriched, with altered levels of amino acids, nucleosides, and sugars. These coordinated changes in diterpenoid metabolism and ABC transporter activity may contribute to the plant’s PEG-simulated drought stress response, though direct functional links between the two pathways require further investigation.

## 4. Discussion

### 4.1. Effects of TaWRKY65-3B Overexpression on Growth and Yield in Rice

WRKY (WRKY domain-containing) transcription factors play pivotal roles in plant growth and abiotic stress responses. They specifically bind to the W-box in the promoter regions of target genes via their conserved WRKY domains, thereby orchestrating the transcriptional regulation of downstream stress-responsive genes [45,46]. They also play indispensable roles in integrating growth and response to abiotic stress, coordinating abscisic acid and reactive oxygen species signaling pathways, and regulating ion homeostasis and osmotic balance [47]. Although *Arabidopsis* quintuple mutants *wrky11/15/17/21/39* and *wrky7/11/17/21/39* exhibited enhanced drought tolerance, this was accompanied by severe growth penalties, including shortened roots, reduced biomass, and delayed flowering [48]. *OsWRKY11* acts as a positive modulator of flowering at relatively low levels. When moderately induced by drought, *OsWRKY11* expression is moderately increased, facilitating heading by directly enhancing the transcription of *OsMADS14* and *OsMADS15*, thereby assisting rice in achieving drought escape [49]. Overexpression of *IlWRKY22* enhances drought tolerance and delays flowering in transgenic *Arabidopsis* [50]. In wheat, the expression of *TaWRKY1* was significantly upregulated under PEG-simulated drought stress, and its overexpression could enhance drought tolerance in transgenic *Arabidopsis* by strengthening ABA signaling and antioxidant capacity [51].

In this study, the constitutive overexpression of *TaWRKY65-3B* in rice resulted in pronounced growth retardation, characterized by reduced plant height, smaller grain size, decreased spikelet number per panicle, and lowered 1000-grain weight, ultimately leading to a significant reduction in grain yield. These phenotypic alterations suggest that *TaWRKY65-3B* functions as a negative regulator of plant growth and reproductive development when ectopically expressed in a heterologous monocot system. Prior research has linked WRKY transcription factors to plant dwarfism. For instance, excessive activity of *OsWRKY36* and *ZmWRKY114* inhibits internode elongation and cell expansion by repressing gibberellin (GA) biosynthesis or stabilizing the DELLA protein SLR1 [52,53]. Similarly, the reduced grain size and diminished thousand-grain weight in *TaWRKY65-3B*-overexpression lines imply that *TaWRKY65-3B* may interfere with hormone signaling pathways-particularly GA, brassinosteroid (BR), and auxin-or indirectly suppress key grain-size regulators (e.g., GW2, GW5/qSW5, GS3, GL3.1/qGL3) involved in lemma-palea cell proliferation and elongation, as documented for other growth-repressing WRKY TFs that modulate the GW5-WRKY53-SGW5 module [54,55,56,57,58]. The concomitant decline in spikelet number per panicle further indicates a possible influence of *TaWRKY65-3B* on panicle branching and inflorescence architecture, likely through crosstalk with the LAX, MOC, or DEP gene networks. Biologically, this growth-yield penalty reflects the well-documented growth-defense/stress-adaptation trade-off: *TaWRKY65* is natively deployed in wheat to enhance drought tolerance-partly via directly promoting the expression of the ethylene biosynthesis gene *TaACS* to modulate ethylene production [59] and reinforcement of stress-responsive gene networks, which, under constitutive high-level expression in the absence of stress, diverts resources from vegetative growth and reproductive investment toward basal defense priming. The yield penalty observed here therefore reinforces that WRKY-mediated stress-adaptive programs, while beneficial under adverse conditions, can be detrimental to agronomic productivity when unregulated. From an applied perspective, these findings caution against the indiscriminate constitutive overexpression of stress-inducible WRKY genes in crop improvement programs and highlight the importance of employing stress-inducible promoters or CRISPR-based allele editing to fine-tune WRKY activity, preserving stress resilience without compromising yield potential.

### 4.2. Diterpenoid Biosynthesis and Metabolic Reprogramming Under PEG-Simulated Drought Stress

Integrated transcriptomic and metabolomic analyses revealed that *TaWRKY65-3B* overexpression in rice under PEG-simulated drought stress significantly upregulates the expression of *OsCPS2* (encoding ent-copalyl diphosphate synthase) and *OsCPS4* (encoding syn-copalyl diphosphate synthase), as well as *OsKS4* (also annotated as *OsKSL4*, encoding syn-pimara-7,15-diene synthase), which catalyze the initial cyclization steps of the labdane-related diterpenoid phytoalexin backbone from geranylgeranyl diphosphate (GGPP) [60,61]. The concomitant up-regulation of *OsCYP76M7*, a cytochrome P450 monooxygenase that catalyzes C11α-hydroxylation of ent-cassadiene to produce phytocassane-type diterpenoid phytoalexins [62,63], together with the induction of *OsCPS2*, *OsCPS4*, and *OsKS4*, indicates activation of the rice phytocassane biosynthetic pathway. Consistent with this transcriptional reprogramming, a diterpenoid metabolite was annotated as neoabietic acid based on MS/MS spectral matching; however, given the well-established role of these genes in phytocassane biosynthesis, this metabolite may represent a phytocassane-type compound or a structurally related labdane-related diterpenoid. Definitive structural identification awaits confirmation with authentic standards. This metabolic shift is consistent with the established paradigm that WRKY transcription factors, such as *OsWRKY10* and *OsWRKY45*, directly bind W-box elements in the promoters of diterpenoid biosynthetic gene clusters (e.g., *OsCPS2*, *OsCPS4*, *OsKSL7*, *OsCYP76M7*) to activate phytoalexin production under stress [64,65]. Biologically, the *TaWRKY65-3B*-mediated reinforcement of the diterpenoid phytoalexin pathway under drought reflects an adaptive chemical defense strategy: the enhanced synthesis of antimicrobial/antioxidant diterpenoids (momilactones, phytocassanes, oryzalexins) helps mitigate drought-induced oxidative damage and potential opportunistic pathogen infection, thereby contributing to cellular homeostasis during water-deficit conditions [66]. The observed co-upregulation of genes from both the momilactone (chromosome 4 cluster: *OsCPS4-OsKSL4-CYP99A2/3*) and phytocassane (chromosome 2 cluster: *OsCPS2-OsKSL7-CYP76M7/M8*) biosynthetic branches suggests that *TaWRKY65-3B* may function as a broad-spectrum activator of rice diterpenoid biosynthetic gene clusters. This extends the known role of wheat WRKYs in abiotic stress adaptation to the transcriptional reprogramming of specialized metabolism in heterologous monocot systems. Notably, the diterpenoid metabolite that accumulated in OE lines—annotated as neoabietic acid based on MS/MS spectral matching—may represent a downstream product or a structurally related labdane-type compound within this activated network, though definitive structural confirmation requires authentic standards.

Notably, the transcriptomic and metabolomic analyses were performed on OE15 as a representative line, selected based on consistent phenotypic trends observed between two independent transgenic lines under PEG-simulated drought stress. However, validation of the key findings—particularly the diterpenoid biosynthetic gene upregulation and metabolite accumulation—in additional independent lines remains necessary to confirm the generality of these results. Furthermore, while the coordinated upregulation of diterpenoid biosynthetic genes and accumulation of a diterpenoid metabolite annotated as neoabietic acid suggest a causal link, direct evidence for transcriptional regulation by *TaWRKY65-3B* is lacking. Further investigation into whether *TaWRKY65-3B* directly binds W-box motifs within the promoters of *OsCPS2*, *OsCPS4*, or *OsCYP76M7* will clarify its regulatory mode (e.g., direct activator vs. co-activator in concert with endogenous OsWRKYs) and help resolve the exact biosynthetic route leading to this metabolite. Whether *TaWRKY65-3B* functions as a direct transcriptional activator or as a co-activator interacting with endogenous rice WRKY proteins remains to be determined.

## 5. Conclusions

In this study, constitutive overexpression of *TaWRKY65-3B* significantly reduced plant height, number of grains per panicle, grain length, grain width, grain perimeter, grain surface area, and thousand-grain weight, ultimately resulting in a reduction in grain yield. However, PEG-simulated drought stress responsiveness was reprogrammed, concomitant with the accumulation of a diterpenoid metabolite annotated as neoabietic acid based on MS/MS spectral matching. This metabolic shift was associated with the up-regulation of key diterpenoid biosynthetic genes, including *OsCPS2*, *OsCPS4*, *OsKS4*, and *OsCYP76M7*, suggesting activation of the rice phytocassane/momilactone biosynthetic network. The precise molecular pathways through which *TaWRKY65-3B* regulates diterpenoid phytoalexin biosynthesis warrant further in-depth investigation.

## Figures and Tables

**Table 1:** Primers used in this study.

Primers	Accession Number	Database	Sequences (5′-3′)	Usage
TaWRKY65-3B-qF	*TraesCS3B02G491100*	Ensembl Plants (IWGSC RefSeq v1.1)	CATCGAGGTGCACGACGAT	For expression of *TaWRKY65-3B*
TaWRKY65-3B-qR			GCGGCCGTGTCGACCTC	
TaActin-qF	*TraesCS2D02G491100*		CTCCCTCACAACAACAACCGC	
TaActin-qR			TACCAGGAACTTCCATACCAAC	
OsActin-qF	*LOC_Os03g50890*	MSU Rice Genome Annotation Project (RGAP v7)	AGTAGTCTCGCCCTGCTTCA	
OsActin-qR			TTCGCCACTTTGTTCTCAACT	
TaWRKY65-3B-163GFP-F	*TraesCS3B02G491100*	Ensembl Plants (IWGSC RefSeq v1.1)	GAGAGGACAGCCCAAGCTTATGGACGGCGAGTGGAGC	For the subcellular localization
TaWRKY65-3B-163GFP-R			CCCTTGCTCACCATGGATCCCGTCATGGCTGTGCCCTC	
TaWRKY65-3B-1390F			CCAAATCGACTCTAGAATGGACGGCGAGTGGAGC	For the transgenic rice
TaWRKY65-3B-1390R			TGCTCACCATGGTACCCACTCACGTCATGGCTGTGCC	

## Data Availability

The RNA-seq data in this study have been deposited in the NCBI Sequence Read Archive (SRA) under BioProject accession PRJNA1497664. The metabolomics data have been deposited in MetaboLights under accession MTBLS15518.

## Supplementary Materials

The following supporting information can be downloaded at https://www.mdpi.com/article/10.3390/antiox15101262/s1. Table S1: The accession numbers of all WRKY protein sequences used in the research.
